# Financial asset allocation duality and enterprise upgrading: empirical evidence from the Chinese A-share market

**DOI:** 10.1057/s41599-023-01683-1

**Published:** 2023-05-15

**Authors:** Ke Guo, Xuemeng Guo, Jun Zhang

**Affiliations:** 1 School of Economics and Management, Beijing Jiaotong University, Beijing, China; 2 School of Accountancy, Beijing Wuzi University, Beijing, China

**Keywords:** Business and management, Finance

## Abstract

This study selects the financial data of Chinese non-financial listed companies from 2012 to 2021 as the research sample and empirically examines in detail the impact of financial asset allocation on enterprise upgrading and its mechanism. The study finds that financial assets have a dual influence on enterprise upgrading. Short-term financial assets provide the necessary funds for production activities, thus promoting enterprise upgrading. Long-term financial assets crowd out the funds needed for production activities and thus inhibit enterprise upgrading, resulting in an inverted U-shaped relationship between financial assets and enterprise upgrading. Mechanism testing revealed that risk-taking capacity and earnings persistence are important ways in which financial assets affect enterprise upgrading. In addition, the impact of financial assets on enterprise upgrading differs for different types of financial assets. The financial asset significantly impacts the upgrading of over-indebtedness, non-state-owned, and high financing constraints enterprises. This study enriches the research literature on financial assets and enterprise upgrading and provides new micro evidence for understanding the impact of financial assets on the enterprise upgrading of listed companies.

## Background

In the “post-subprime crisis” economic development transition period, the entity industry market demand is saturated and declining. To achieve the purpose of capital appreciation, most companies allocate capital to the financial sector “idle,” resulting in the continued financialization of investment methods and income channels. The continued financialization of the real economy has become a microscopic feature of macroeconomic development in many countries and regions worldwide, including China. The outbreak of global COVID-19 has caused the real economy to stagnate. The trend toward the financialization of the economy has intensified. To make matters worse, the lack of input growth in the real economy has led to problems that have made it difficult for the enterprise to upgrade. This poses a severe challenge to economic recovery on a global scale.

From a micro perspective, unblocking deep financialization, eliminating the development dilemma of physical circulation, and promoting the upgrading of entity enterprises, are issues that entity enterprises need to address at this stage. From a macro perspective, addressing the market bubbles is essential to achieving a rapid economic recovery in developing countries represented by China. The above is related to corporate financialization. Thus, exploring the microeconomic consequences of corporate financialization and promoting financial services to the real economy has become a hot topic for governments and academics. Since its inception, the concept of “corporate financialization” has been discussed in academic circles. Stockhammer (2004) offers a much more comprehensive account of financialization, which encompasses financial market globalization, shareholder revolutions, and increased financial revenue. Baud and Durand (2012) propose three dimensions that distinguish financialization: first, the increase in the volume and frequency of financial flows from entity firms to the financial sector; second, the increase in the number of financial assets allocated by entity firms; and third, the support that entity firms provide to their customers or suppliers for the financial activity to obtain long-term permission from customers and suppliers. This paper focuses on micro-level financialization, i.e., corporate financialization. With the increasing prosperity of the financial market, the academic community has extensively studied the financialization of firms. It has become a research hotspot in recent years. Studies have been conducted to explore corporate financial asset allocation, both influencing factors and economic consequences. Macroeconomic factors, such as the GDP business cycle, the money supply, economic policy uncertainty, environmental regulations, industrial policy, and tax policy, all affect companies’ allocation of financial assets (Chaney, 2016; Riccetti et al., 2016; Kim and Kung, 2017; Ziaei, 2022; Xie et al., 2022). Macroeconomic factors, such as institutional investors, operating income, financing constraints, heterogeneity of executive characteristics, social responsibility commitment, equity pledges, and performance commitments, all have positive or negative effects on corporate financial asset allocation (Edmans et al., 2018; Ferreira et al., 2019).

Regarding economic consequences, existing studies focus on the “reservoir effect” and the “crowding out effect.” The “reservoir effect” considers financial assets to be flexible, reversible, and highly liquid, which enables companies to deploy funds promptly to diversify the risk of external shocks and ensure liquidity security when internal cash flow is tight (Sen and Dasgupta, 2018; Barane and Hake, 2018; Zhu and Xiao, 2022). Besides, financial assets can broaden the financing channels of enterprises and relieve the pressure of reliance on external financing, thus promoting the production activities of enterprises (Duchin et al., 2017; Su and Liu, 2021). The “crowding out effect” considers the economic consequences of financial asset allocation at both the macro and micro levels. At the macro level, the rise in financial assets will increase macro leverage and exacerbate the degree of asset misallocation. This will lead to a continued expansion of the virtual economy and may even bring the real economy to a standstill (Allen et al., 2019; Liu et al., 2022). At the micro level, the impact of the increase in financial assets on enterprises is reflected through the crowding out of physical investment and R&D, lowering audit quality, raising the cost of debt capital, and reducing labor productivity (Du et al., 2017; Tori, Onaran, 2018; Hahn et al., 2019; Liu et al., 2022; Jin et al., 2022). As we can see, most academic empirical studies on financial asset allocation have focused on the linear relationship. However, there is a lack of necessary exploration of the effect of financial assets on the upgrading of enterprises at the meso level, the mechanism of action, and the heterogeneity effect.

Based on the fact that different financial assets have both positive and negative characteristics. This study takes enterprise upgrading as the starting point to explore the microeconomic consequences, impact mechanisms, and heterogeneity effects of allocating financial assets in the context of China’s economic transition. This paper tries to answer the following related academic questions: (1) What effects would financial asset allocation exert on the enterprise upgrading process? (2) What micro-mechanisms affect this impact? (3) Does this effect vary depending on the company? Compared with existing studies, the main contribution of this study is reflected in the following aspects.

First, it is an essential supplement to research on the microeconomic consequences of corporate financial asset allocation. This study is a beneficial enrichment of the relevant literature. We selected the research perspective of enterprise upgrading, introduced its development logic into the analytical framework, verified the dual effect of corporate financial allocation, and proposed the theoretical hypothesis of a nonlinear relationship between financial asset allocation and enterprise upgrading. After a series of robustness tests, such as the substitution of critical indicators, one-period lagged regression, and controlling for province-fixed effects, after a series of robustness tests, this study verifies the conclusion that there is a nonlinear relationship between financial asset allocation and enterprise upgrading. Second, this study empirically tests whether the risk-taking capacity and the earnings persistence are essential for financial assets to affect the enterprise upgrading of A-share listed companies by constructing the corresponding intermediation effect model. We clarify the micro-level mechanisms of the impact of long-term and short-term financial assets on enterprise upgrading and reveal the dynamic pattern of this impact, which is a necessary addition to the existing literature on both. The analysis of the influence mechanism is evidently conducive to deepening the understanding of the relationship between financial assets and enterprise upgrading. Third, we examine the differences in the effects of financial asset allocation in different types of tight groupings. Considering that corporate allocation to financial assets is conditional, this study also analyzes the heterogeneous impact of financial assets on the enterprise upgrading of different types of enterprises (over-indebted and non-over-indebted enterprises, ownership types, and degree of financing constraints). This study provides a rich perspective for a comprehensive understanding of the enterprise upgrading of financial assets by exploring the differences in the impact of financial assets on the enterprise upgrading of different types of enterprises. It also provides empirical evidence and a reference basis for enterprises’ targeted allocation of financial assets.

The remainder of this paper is structured as follows: Section “Theoretical analysis and research hypothesis” develop research hypotheses. The “research design” section describes samples, data, measures, and statistical techniques. Section “Data analysis and discussion” outlines our empirical results. Section “Further test” describes the heterogeneity test and moderating effect test. Finally, concludes, discusses the policy implications, limitations, and future research directions are summarized in the Section “Conclusion.”

## Theoretical analysis and research hypothesis

The issues surrounding China’s economic transformation are gradually coming to light, and the contradictions between supply-side structural reform and social development are becoming increasingly apparent. The unique institutional context and economic realities provide the conditions for the emergence of financial bubbles in capital markets. As a result, industrial capital has achieved economies of scale, and the marginal benefits of physical production activities are diminishing, leading to the emergence of financial investments (Gulen and Ion, 2016). This study classifies firms’ motives for investing in financial assets as cash reserves and capital arbitrage and explores the dual economic consequences of these different motives.

### Cash reserve motivation

The impact of the cash reserve motive is primarily reflected in short-term financial assets. Firstly, short-term financial assets have a shorter liquidation period, are more convenient to circulate, and are traded by holders for various purposes. Therefore, short-term financial assets can achieve the futures hedging and risk aversion holders wish to achieve. Investing in short-term financial assets can play a vital role in a company’s cash flow, serving as a precautionary saving measure and smoothing potential financial risks. The flexibility and liquidity of short-term financial assets can relieve the high cost of external investment and financing activities, facilitate the smooth circulation and efficient turnover of internal funds, relieve capital pressure, optimize the financing structure, and improve the efficiency of financing. This, in turn, promotes the upgrading of enterprises at the capital level (Gehringer, 2013; Ding et al., 2013). Secondly, sufficient capital reserves are prerequisites for sustainable research and development (R&D) activities. When faced with financial constraints or the risk of breaking the capital chain, enterprises’ R&D (research and development) activities will be forced to a standstill, seriously damaging physical production activities and thus inhibiting the enterprises’ upgrading. Flexible, short-term financial assets can help companies promptly expand external financing channels, effectively bridge the financing gap, and provide necessary financial support for R&D activities such as technology development, human resource optimization, and production efficiency improvement. This, in turn, promotes the upgrading of enterprises at the technology iteration and efficiency improvement level (Chang et al., 2017; Davis, 2018; Hou et al., 2021). Finally, short-term financial assets are easier to trade, with shorter turnarounds than physical production activity and a relatively higher yield. High returns on financial assets in the short term can improve the balance sheet position, increase corporate profitability, and provide incremental space for future growth. Therefore, short-term financial assets can enhance a company’s ability to survive in an environment of uncertainty, promoting the upgrading of enterprises at the enterprise operating conditions level (Soener, 2015).

Based on the above analysis, the following hypothesis is proposed:

*Hypothesis 1 Short-term financial assets have positive impacts on enterprise upgrading*.

### Capital arbitrage motivation

The capital arbitrage motive primarily manifests itself in long-term financial assets. On the one hand, long-term financial assets, such as investment properties, are characterized by significant investment amounts, lengthy amortization periods, and significant investment fluctuations. As a result, investors often see them as an arbitrage opportunity. The excess profits can create a vicious cycle of “long-term financial asset investment—gaining excess returns—reinvestment in financial assets,” leading to the deviation of production and operation sequences. This leads to the capital’s withdrawal from the principal-business side and a shift to the financial asset investment side. This results in a reduction in enterprise willingness to invest and the proportion of capital expenditure, increased difficulty in industrial accumulation, lower production efficiency levels, and diminished ability to carry out R&D activities. Ultimately, this hinders the upgrading of enterprises. Some individual companies may even take advantage of this market capitalization by investing in long-term financial assets using financial leverage, despite a lack of internal capital. However, such behavior may exacerbate the risk of over-indebtedness, intensify the contradiction of “de-industrialization” in the market, and spawn several asset bubbles (González and Sala, 2014; Breeden and Viswanathan, 2015; Hasan et al., 2022).

On the other hand, the progressive increase of the long-term financial asset component in the total asset component of an enterprise may cover the short-term drop in income from the physical production business. However, its long-run irreversible characteristics make it challenging to have a flexible turnover of funds. Corporate development becomes dependent on fluctuations in the prices of related financial products and derivatives. Many factors constrain the long-term development of enterprises, preventing them from achieving transformation and upgrading. Over-reliance on long-term financial assets may increase the risk of disruptions to internal cash flow. The decline in investment in real assets, such as renewed fixed assets and refurbishments, leads companies to find it hard to obtain external credit. As a result, it becomes increasingly difficult for companies to obtain long-term loans through collateral and other financing methods, which increases the maturity mismatch of funds. Under these circumstances, the investment and income of corporations are in a highly precarious state, and cash flow fluctuations are highly uncertain. Once the accumulation of financial risks occurs, it will lead to a series of chain reactions, such as significant depreciation of real assets and excessive liabilities, making daily production unsustainable and eventually hindering the upgrading of enterprises (Gleadle et al., 2014; Aghion et al., 2019; Lee et al., 2020).

Based on the above analysis, the following hypothesis is proposed:

*Hypothesis 2 Long-term financial assets have negative impacts on enterprise upgrading*.

### Combining the motivations

In summary, the “cash reserve” significantly promotes the upgrading of enterprises. Due to their excellent liquidity, flexibility, and reversibility, short-term financial assets can promptly cover the capital gap between production and investment and relieve enterprises’ financial distress, thus achieving the purpose of preventive reserve. “Capital arbitrage” significantly disincentivizes enterprise upgrading. Long-term financial assets are challenging to turn around flexibly, have long payback periods, require considerable investment amounts, and often have a specific crowding-out effect on a company’s physical production needs. Evidently, investing in different types of financial assets has opposite effects on enterprise upgrading, i.e., financialization can hinder or promote it. As investment in long-term financial assets is not a relatively isolated process from investment in short-term financial assets, the positive effects that short-term finance can have on companies may induce them to make many blind investments in long-term financial assets. Furthermore, overinvestment is more common in long-term financial assets, and there is a time lag between the effect of such overinvestments on firm upgrading. An analysis of the superimposed effects of long-term and short-term financial assets on the corporate upgrading situation concludes that a complicated nonlinear relationship exists between financial asset allocation and enterprise upgrade, whereby the impact of financial assets on corporate upgrading has a dual effect.

Based on the above analysis, the following hypothesis is proposed:

*Hypothesis 3 Financial assets and enterprise upgrading show an inverted U-shaped relationship*.

## Methodology

### Data and sample

To ensure that time series data remain intact while also considering the time lag of an enterprise’s upgrade, the data of this study covers Chinese non-financial A-share listed companies from 2012 to 2021. We collect data from several resources. Financial data of listed companies are obtained from the China Securities Markets and Accounting Research Database, Wind Economic Database, and Juchao Information websites. To ensure the accuracy of the data, we cross-check it with the company’s annual report to verify the authenticity of the data. Exclude samples with unknown or missing critical financial data disclosure, accounting insolvency (LEV > 1), unusual listing status such as ST/*ST/PT during the sample study period, IPO in the year of listing, cross-listing of A/H/N/B shares, and violate accounting common sense such as total assets less than net fixed assets or current assets. To eliminate interferences of exceptionally abnormal values in the research, we have Winsorize of all consecutive type variables in the [1% to 99%] range.

### Operationalization of key variables

#### Dependent variable: financial asset allocation

The existing relevant empirical literature measures the extent of enterprises’ “de-realization” by the ratio of financial assets to total assets or by the percentage of financial assets earnings to net income. Considering that investing in financial assets and earning returns is often a dynamic process, this paper selects the percentage of financial assets held to measure the degree of financialization of firms. Concerning the existing literature, we include monetary fund, trading financial assets, available-for-sale financial assets, held-to-maturity investments, long-term equity investments, and investment property in the category of financial assets (*FIN*) (Demir, 2009; Zhu et al., 2021). Further, in order to distinguish the motives of enterprises holding different financial assets and to analyze the economic consequences of the resulting different effects on the enterprise, based on a difference in the investment maturity and liquidity, we have established that the more liquid monetary fund and trading financial assets are short-term Financial Asset (*FIN_ S*). In contrast, several other financial assets are long-term finance assets (*FIN_ L*).

Considering the changes in China’s ASBEs (Accounting Standards for Business Enterprises) during the study period, the accounts “held-to-maturity investments” and “available-for-sale financial assets” have been removed from the corporate balance sheet after 2018. Therefore, in measuring financial assets after 2018, we use “debt investments” instead of “held-to-maturity investments” and “other debt investments” and “investments in other equity instruments” instead of “available-for-sale financial assets.”

#### Independent variable: enterprise upgrading

Enterprise upgrading is the micro expression of industrial structure upgrading, which typically refers to the process of gradually evolving from labor-intensive to technology-intensive or capital-intensive forms of enterprise production. Specifically, the transformation process of “Original Equipment Manufacturer (OEM)—Original Design Manufacturer (ODM)—Original Brand Manufacturer (OBM).” Existing studies on enterprise upgrading mainly focus on influencing factors and path selection, which effectively measures the transformation efficiency of production factors, but have limitations in identifying the “qualitative change” and “quantitative change” of the upgrading effect. It is not easy to fully express the essence of enterprise upgrading (Kergroach, 2019). Following the established literature that uses productivity to measure industrial structural upgrading, this study selects total factor productivity to measure enterprise upgrading. This method not only considers that the essence of enterprise upgrading is to improve production efficiency and value chain upgrading but also is widely favored by scholars because of its comprehensive information coverage (Liu et al., 2022). To alleviate the endogeneity concern as well as avoid the problem of sample selection bias, we selected two methods: one using the Olley-Pakes method (*OP*) and another using Levinsohn-Petrin’s method (*LP*) to account for the firm’s total factor productivity (Dindial et al., 2020):

1 $$\begin{array}{*{20}{c}} {PE_{j,t} = TEP_ - OP_{j,t}\left( {TFP_ - LP_{j,t}} \right) = lnY_{j,t} - \varphi _alnK_{j,t} - \varphi _blnL_{j,t}} \end{array}$$

In Eq. (1), *PE*_*j,t*_ represents the production efficiency considering the relationship between intermediate input and total output value, which is used to reflect the final production capacity of the enterprise; *Y*_*j,t*_ represents the main business income (Giannetti et al., 2015); *K*_*j,t*_ represents the fixed assets; and *L*_*j,t*_ represents the number of employees of the enterprise.

The production function of the OP method is (Olley, Pakes, 1996):

2 $$\begin{array}{*{20}{c}} {lnY_{j,t} = \varphi _0 + \varphi _alnK_{j,t} + \varphi _blnL_{j,t} + \varphi _cAge_{j,t} + \varphi _dState_{j,t} + \varphi _EEX_{j,t}} \\ { + {\sum} {\varphi _eYear_e} + {\sum} {\varphi _fReg_f} + {\sum} {\varphi _gInd_g} + \varepsilon _{j,t}} \end{array}$$

*Age*_*j,t*_ represents the year of listing; *State*_*j,t*_ represents the dummy variable for the nature of enterprise ownership; *EX*_*j,t*_ represents the dummy variable for whether the enterprise is involved in export activities; *Year*_*e*_, *Reg*_*f*_, *Ind*_*g*_ represents the year, region, and industry respectively; state variable(*STATE*) are *InK*_*j,t*_ and *Age*_*j,t*_; control variables (*CVARS*) are *State*_*j,t*_ and *EX*_*j,t*_; exit variables (*EXIT*), which is determined by the existence of one of the following circumstances: (i) whether the enterprise is delisted during the study period, (ii) whether the enterprise is listed in a shell during the study period, and (iii) whether the enterprise has changed its business and main business during the study period; all other variables are free variables (*FREE*).

The LP method production function is (Levinsohn and Petrin, 2003):

3 $$\begin{array}{*{20}{c}} {lnY_{j,t} = \varphi _0 + \varphi _alnK_{j,t} + \varphi _blnL_{j,t} + \varphi _clnM_{j,t} + \varepsilon _{j,t}} \end{array}$$

The LP method is based on the OP method, where intermediate inputs (*M*_*j,t*_) are chosen as proxies to control for shocks to productivity from unobservable factors. In this research paper, intermediate inputs are measured in terms of actual cash paid by firms through the purchase of goods and services received.

#### Definition of main variables

Similarly, following previously published studies, we introduce the following control variables in this study: capital structure (*LEV*), cash flow (*CFO*), profitability (*ROE*), capital intensity (*TAG*), asset size *(SIZE*), investment opportunity (*GROW*), labor intensity (*LAB*), and years on the market (*AGE*) were selected as control variables. The variables are defined in Table 1.

**Table 1:** Definition of main variables.

Variable	Symbol	Definition
Financial asset allocation	*FIN*	Financial assets/total assets
Short-term financial asset allocation	*FIN_S*	Short-term financial assets/total assets
Long-term financial asset allocation	*FIN_L*	Long-term financial assets/total assets
Enterprise upgrade	*TEP_OP*	See Eqs. (1) and (2)
	*TEP_LP*	See Eqs. (1) and (3)
Capital structure	*LEV*	Total liabilities/total assets
Cash flow	*CFO*	Operating cash flow/total assets
Profitability	*ROE*	Net income/average shareholders’ equity
Capital intensity	*TAG*	Net fixed assets/total assets
Asset size	*SIZE*	LN (total assets)
Investment opportunities	*GROW*	Sales revenue growth rate
Labor intensity	*LAB*	Cash paid to and for employees/total operating revenues
Number of years on the market	*AGE*	Ln (year of observation – the year of release)

#### Empirical specification

To analyze the impact of Short-term Financial Asset and Long-term Financial Asset Allocation on the Enterprise Upgrade of China Listed Companies, this study sets the following benchmark regression model:

4 $$\begin{array}{*{20}{c}} {TEP_ - OP_{j,t}\left( {TFP_ - LP_{j,t}} \right) = \alpha _0 + \alpha _1FIN\_S_{j,t}\left( {FIN\_L_{j,t}} \right) + \gamma {\it{Control}}_{j,t} + \Sigma \eta _{j,t} + \Sigma \mu _{j,t} + \varepsilon _{j,t}} \end{array}$$

*α*_0_ represents the intercept term of the model; *Control*_*j,t*_ represents the control variable. To overcome the endogeneity problem caused by the omitted variables, *η*_*j,t*_ and *μ*_*j,t*_ are the time-individual two-way fixed effects used in this study; *ε*_*j,t*_ represents the random disturbance term.

To verify the nonlinear relationship between financial asset allocation and enterprise upgrading, this study sets up the following regression model:

5 $$\begin{array}{*{20}{c}} {TEP_ - OP_{j,t}\left( {TFP_ - LP_{j,t}} \right) = \alpha _0 + \alpha _1FIN_{j,t} + \alpha _2FIN\_SQ_{j,t} + \gamma {\it{Control}}_{j,t} + \Sigma \eta _{j,t} + \Sigma \mu _{j,t} + \varepsilon _{j,t}} \end{array}$$

*FIN*_*SQ*_*j,t*_ is a quadratic term representing *FIN*_*j,t*_. Referring to the existing studies on the nonlinear relationship, we test the inverted U-shaped relationship mainly based on the following three fronts: (1) *α*_2_ < 0; (2) *α*_1_ + 2*α*_2_*FIN*_*MIN*_ > 0, *α*_1_ + 2*α*_2_*FIN*_*MAX*_ < 0; (3) the inflection point falls within the interval of the independent variables’ values.

We tested the variables included in the model for multicollinearity, and the Pearson correlation coefficients between the variables showed no significant multicollinearity. The statistical regressions in this study use a fixed effects model, with standard errors adjusted for clustering and robust adjustment at the firm level.

## Results

### Summary statistics

Table 2 displays the descriptive statistics of the variables examined in the initial assessment. The mean value of *FIN* is 0.221, with the minimum and maximum values being 0.020 and 0.728, respectively. These figures suggest that non-financial Chinese A-share listed companies generally tend to allocate financial assets, with a relatively significant difference in the number of assets observed. The average values of *FIN_S* and *FIN_L* are 0.193 and 0.027, respectively, signifying that the allocation of short-term financial assets is the primary characteristic of corporate financialization. The statistical values of *TEP_OP* are near to those of *TEP_LP*, and all the statistical values are slightly lower than *TEP_LP*, aligning with theoretical expectations. The distribution of the remaining variables is primarily consistent with prior published studies.

**Table 2:** Summary statistics.

Variable	*N*	Mean	Std. dev.	Min	Median	Max
*FIN*	9640	0.221	0.152	0.020	0.178	0.728
*FIN_S*	9640	0.193	0.141	0.013	0.152	0.677
*FIN_L*	9640	0.027	0.062	0	0.002	0.386
*TEP_OP*	9640	6.673	0.890	4.593	6.552	9.033
*TEP_LP*	9640	6.854	0.902	4.776	6.736	9.232
*LEV*	9640	0.430	0.217	0.046	0.423	0.943
*CFO*	9640	0.043	0.070	−0.186	0.042	0.234
*ROE*	9640	0.068	0.111	−0.482	0.071	0.344
*TAG*	9640	0.229	0.168	0.002	0.196	0.721
*SIZE*	9640	21.990	1.266	19.511	21.812	25.876
*GROW*	9640	−0.056	0.310	−0.801	−0.081	1.446
*LAB*	9640	0.126	0.088	0.010	0.106	0.505
*AGE*	9640	2.799	0.406	1.946	2.833	3.401

### Baseline results

Table 3 exhibits the regression results of financial asset allocation and enterprise upgrading. Columns (1)–(2) demonstrate that the estimated coefficient of short-term financial asset allocation(*FIN_S*) on the Olley-Pakes Method of total factor productivity (*TEP_OP*) and Levinsohn-Petrin Method of total factor productivity (*TEP_LP*) is positive and significant, signifying that with the rise of short-term financial assets, enterprises will also experience a significant upgrade. Columns (3)-(4) indicate that long-term financial asset allocation (*FIN_L*) is positively related to *TEP_OP* and *TEP_LP* at the 1% significance level, implying that long-term financial asset allocation impedes substantial upgrading; Hypotheses 1 and 2 are thus confirmed.

**Table 3:** Baseline effects test: financial asset and enterprise upgrading.

Variable	(1)	(2)	(3)	(4)	(5)	(6)
	*TEP_OP*	*TEP_LP*	*TEP_OP*	*TEP_LP*	*TEP_OP*	*TEP_LP*
*FIN_S*	0.117^*^	0.117^*^				
	(1.862)	(1.857)				
*FIN_L*			−0.594^***^	−0.558^***^		
			(−3.771)	(−3.529)		
*FIN*					0.292^**^	0.312^**^
					(2.036)	(2.179)
*FIN_SQ*					−0.525^***^	−0.542^***^
					(−2.697)	(−2.798)
*LEV*	0.299^***^	0.278^***^	0.258^***^	0.239^***^	0.268^***^	0.249^***^
	(5.912)	(5.503)	(5.312)	(4.909)	(5.254)	(4.887)
*CFO*	0.480^***^	0.438^***^	0.519^***^	0.478^***^	0.532^***^	0.487^***^
	(4.846)	(4.415)	(5.460)	(5.005)	(5.387)	(4.919)
*ROE*	0.572^***^	0.548^***^	0.563^***^	0.540^***^	0.565^***^	0.541^***^
	(8.916)	(8.547)	(8.831)	(8.461)	(8.824)	(8.459)
*TAG*	−0.616^***^	−0.456^***^	−0.689^***^	−0.527^***^	−0.655^***^	−0.491^***^
	(−9.308)	(−6.902)	(−10.959)	(−8.379)	(−9.585)	(−7.202)
*SIZE*	0.359^***^	0.371^***^	0.358^***^	0.369^***^	0.359^***^	0.370^***^
	(42.964)	(44.308)	(43.166)	(44.510)	(43.180)	(44.550)
*GROW*	0.070^***^	0.072^***^	0.075^***^	0.076^***^	0.072^***^	0.073^***^
	(3.740)	(3.811)	(4.000)	(4.062)	(3.841)	(3.907)
*LAR*	−4.538^***^	−4.623^***^	−4.525^***^	−4.611^***^	−4.502^***^	−4.587^***^
	(−32.729)	(−33.064)	(−32.729)	(−33.071)	(−32.382)	(−32.698)
*AGE*	0.048^**^	0.049^**^	0.068^***^	0.068^***^	0.046^**^	0.047^**^
	(2.070)	(2.102)	(2.948)	(2.915)	(2.012)	(2.042)
*η/μ*	YES	YES	YES	YES	YES	YES
*_cons*	−1.133^***^	−1.225^***^	−1.090^***^	−1.182^***^	−1.121^***^	−1.218^***^
	(−6.109)	(−6.606)	(−6.007)	(−6.516)	(−5.984)	(−6.503)
*adj R*^*2*^	0.776	0.781	0.777	0.782	0.776	0.781
*N*	9640	9640	9640	9640	9640	9640

***, **, * represents significance at the 1%, 5%, and 10% levels, respectively, with *Z*-values in parentheses.

Columns (5)–(6) show that the financial asset (*FIN*) is positively correlated with *TEP_OP* and *TEP_LP*. In contrast, its quadratic term (*FIN_SQ*) negatively correlates with *TEP_OP* and *TEP_LP* at the 1% significance level. After separate calculations, the inflection points of *FIN_SQ* with *TEP_OP* and *TEP_LP* to form the inverted U-shaped curve are 0.278 and 0.288, respectively, both within a reasonable range of the sample values. The slope has a different sign before and after the extreme value of the curve^1^. This demonstrates a threshold increase in financial assets for the enterprise to be upgraded, so if the threshold is exceeded, financial assets would be a hindrance to upgrading the enterprise. The UTEST test on the two curves in Eq. (5) yields *t*-values of 1.99 and 2.13, respectively, and both pass the significance test of 5%. The above results illustrate an inverted “U” shaped relationship between the increase in financial assets and enterprise upgrading, thereby verifying Hypothesis 3.

As shown in Table 2, the mean value of *FIN* is 0.221, which has exceeded the inflection point, indicating that the effect of financial assets on enterprise upgrading of the Chinese A-share listed is on the right side of the inverted “U” curve. Excessive investment in financial assets has already weakened the upgrades of some A-share companies.

### Robustness checks

To ensure the robustness of the study, we further conducted the following tests: (1) Replacement of the dependent variable. *TFP* was re-measured using Ordinary Least Square (*OLS*) method based on sample data; the new dependent variable was lagged by one period and then regressed to alleviate possible contemporaneous endogeneity concerns. (2) One-period lagged regression. Given the potential lagged effect of investment in financial assets on enterprise upgrading and to avoid the contemporary related endogeneity problem, we regress the dependent variable in the benchmark model with a one-period lag. (3) Controlling for province-fixed effects (*PROV*). To control for the influence of region-level factors that do not change over time on the study results, we include the province-fixed effect of the firm’s registered address in the benchmark model.

We run the regression using the alternative measures and with the same set of control variables as those. The above steps were repeated for the empirical regression analysis of the original model, and the specific results are presented in Table 4. Panel A of Table 4, where *TEP_OLS* denotes the Ordinary Least Square Method of total factor productivity, as Panel A of Table 4 reveals that the association between financial assets and enterprise upgrading remains significant. In Panel B of Table 4, L.*TFP_OP* and *L.TFP_LP* denote the one-period lag of *TFP_OP* and *TFP_LP*, respectively, and the regression results are essentially consistent with the baseline results. In Panel C of Table 4, we added province-fixed effects (*PROV*), and the regression results are still compatible with the baseline results. As seen from Table 4, our results have not changed significantly, affirming this study’s robustness.

**Table 4:** Robustness tests.

Panel A	OLS method re-measures TFP					
	*TEP_OLS*		*TEP_OLS*		*TEP_OLS*	
*FIN_S*	0.187^*^					
	(1.864)					
*FIN_L*			−0.836^***^			
			(−4.063)			
*FIN*					1.327^***^	
					(6.075)	
*FIN_SQ*					−2.222^***^	
					(−7.123)	
*Control*	YES		YES		YES	
*η/μ*	YES		YES		YES	
*_cons*	−7.599^***^		−7.443^***^		−7.767^***^	
	(−25.080)		(−24.876)		(−25.931)	
*Adj R*^*2*^	0.809		0.810		0.812	
*N*	7039		7039		7039	
**Panel B**	**One-period lagged regression of the dependent variable**					
	*L.TFP_OP*	*L.TFP_LP*	*L.TFP_OP*	*L.TFP_LP*	*L.TFP_OP*	*L.TFP_LP*
*FIN_S*	0.233^***^	0.230^***^				
	(2.931)	(2.872)				
*FIN_L*			−0.421^**^ (−2.361)	−0.389^**^		
				(−2.168)		
*FIN*					0.638^***^	0.637^***^
					(3.286)	(3.299)
*FIN_SQ*					−0.902^***^	−0.893^***^
					(−3.099)	(−3.089)
*Control*	YES	YES	YES	YES	YES	YES
*η/μ*	YES	YES	YES	YES	YES	YES
*_cons*	−1.488^***^	−1.598^***^	−1.354^***^	−1.469^***^	−1.542^***^	−1.655^***^
	(−6.157)	(−6.594)	(−5.670)	(−6.140)	(−6.265)	(−6.701)
*Adj R*^*2*^	0.713	0.719	0.713	0.719	0.713	0.719
*N*	7039	7039	7039	7039	7039	7039
**Panel C**	**Controlling for province-fixed effects**					
	*TFP_OP*	*TFP_LP*	*TFP_OP*	*TFP_LP*	*TFP_OP*	*TFP_LP*
*FIN_S*	0.126^**^	0.127^**^				
	(2.063)	(2.070)				
*FIN_L*			−0.806^***^	−0.771^***^		
			(−5.284)	(−5.035)		
*FIN*					0.234^*^	0.254^*^
					(1.708)	(1.862)
*FIN_SQ*					−0.498^***^	−0.515^***^
					(−2.710)	(−2.822)
*Control*	YES	YES	YES	YES	YES	YES
*PROV*	YES	YES	YES	YES	YES	YES
*η/μ*	YES	YES	YES	YES	YES	YES
*_cons*	−0.765^***^	−0.851^***^	−0.700^***^	−0.787^***^	−0.729^***^	−0.820^***^
	(−4.068)	(−4.525)	(−3.828)	(−4.301)	(−3.843)	(−4.324)
*Adj R*^*2*^	0.788	0.792	0.790	0.794	0.788	0.793
*N*	9640	9640	9640	9640	9640	9640

***, **, * represents significance at the 1%, 5%, and 10% levels, respectively, with Z-values in parentheses.

### Influence channel analysis

To fully comprehend the theoretical logic between financial assets and enterprise upgrading, it is necessary to explore the classification of potential mechanisms. Regarding short-term financial assets, when enterprises face financial distress caused by financing and investment, short-term financial assets can revitalize the stock of funds and optimize the structure of capital reserves. This not only ensures the sustainability of cash flow but also mitigates the possible risk of capital breakage, ultimately enhancing the risk-bearing capacity of the enterprise and ensuring the smooth progress of upgrading. As for long-term financial assets, excessive reliance on non-physical production income from long-term financial assets under limited capital reserve will not only exacerbate the conflict between the funds required for business production and the actual cost but also squeeze out the primary business income of enterprises, ultimately reducing the sustainability of surplus and hindering enterprise upgrading.

We adopt risk-taking capacity (*RISK*) as an intermediate variable for short-term financial assets impacting enterprise upgrading and earnings persistence (*EP*) as an intermediate variable for long-term financial assets impacting enterprise upgrading. The modified Altman *Z*-score is used as a proxy variable for enterprise risk-taking ability, and a higher value indicates that the actual financial risk of the enterprise is less significant. The proportion of enterprise sales revenue to the average book value of total assets is utilized as a proxy variable for earnings persistence, where the average book value of total assets is calculated as (opening book value of total assets + closing book value of total assets)/2.

We constructed the following stepwise regression model to test the mechanism:

6 $$\begin{array}{*{20}{c}} {RISK_{j,t}\left( {EP_{j,t}} \right) = \alpha _0 + \alpha _1FIN_ - S_{j,t}\left( {FIN_ - L_{j,t}} \right) + \alpha _2FIN_ - SQ_{j,t} + \gamma {\it{Control}}_{j,t} + } \\ {\Sigma \eta _{j,t} + \Sigma \mu _{j,t} + \varepsilon _{j,t}} \end{array}$$

7 $$\begin{array}{*{20}{c}} {TEP_ - OP_{j,t}\left( {TFP_ - LP_{j,t}} \right) = \alpha _0 + \alpha _1RISK_{j,t}\left( {EP_{j,t}} \right) + \alpha _2FIN_ - S_{j,t}\left( {FIN_ - L_{j,t}} \right) + \gamma {\it{Control}}_{j,t}} \\ { + \Sigma \eta _{j,t} + \Sigma \mu _{j,t} + \varepsilon _{j,t}} \end{array}$$

Table 5 presents the mechanism test results for the impact of short-term and long-term financial assets on enterprise upgrading. In Panel A, *FIN_S* and *RISK* are positively correlated at 1% significance level, indicating that short-term financial assets can increase firms’ risk-taking capacity; after including mediating variables to the Baseline model, the correlation coefficients between *FIN_S* and *TFP_OP* and *TFP_LP* diminish and do not pass the significance test. At the same time, *RISK* maintains significant correlations with *TFP_OP* and *TFP_LP*, respectively, indicating that risk-taking ability is the potential mechanism for short-term financial assets to influence enterprise upgrading.

**Table 5:** Mechanism test.

Panel A	*RISK*	*TFP_OP*	*TFP_LP*
*FIN_S*	4.277^***^	0.098	0.098
	(4.581)	(1.547)	(1.539)
*RISK*		0.007^***^	0.007^***^
		(5.260)	(5.481)
*Control*	YES	YES	YES
*η/μ*	YES	YES	YES
*_cons*	23.987^***^	−1.320^***^	−1.416^***^
	(9.933)	(−6.898)	(−7.399)
*adj R*^*2*^	0.433	0.778	0.783
*N*	9385	9385	9385
**Panel B**	***EP***	***TFP_OP***	***TFP_LP***
*FIN_L*	−0.663^***^	−0.180	−0.165
	(−5.049)	(−1.131)	(−1.021)
*EP*		0.695^***^	0.671^***^
		(28.176)	(26.480)
*Control*	YES	YES	YES
*η/μ*	YES	YES	YES
*_cons*	0.559^***^	−1.627^***^	−1.701^***^
	(3.406)	(−10.249)	(−10.487)
*Adj R*^*2*^	0.385	0.848	0.846
*N*	7036	7036	7036

***, **, * represents significance at the 1%, 5%, and 10% levels, respectively, with Z-values in parentheses.

In Panel B, *FIN_L* is negatively correlated with *EP* at the 1% significance level, indicating that long-term financial assets can impede corporate earnings persistence; after including mediating variables into the Baseline model, the correlation coefficients between *FIN_L* and *TFP_OP* and *TFP_LP* decrease and become insignificant. At the same time, *EP* retains the significant correlation between *TFP_OP* and *TFP_LP*, implying that earnings persistence is a potential mechanism for long-term financial assets to influence enterprise upgrading.

## Extensibility test

The benchmark analysis and correlation test on how financial assets affect enterprise upgrading was conducted above; we point out the dynamic nonlinear relationship between financial assets and enterprise upgrading. In order to identify and explore how the impact of financial assets on enterprise upgrading varies with firm characteristics, this is categorized and explored below.

### Heterogeneity test

#### Liability heterogeneity test

The level of debt determines the cash flow available to the company; for a firm, overleveraging means that the amount of debt financing is more than the firm can afford. We argue that over-indebtedness firms are more likely than non-over-indebtedness firms to use limited debt funds to invest in financial assets in order to generate profits or repay debt. The Tobit regressions in this study are run for the total sample by year and industry. The target leverage ratio of firms (LVB*t) is calculated based on the model’s residuals. If the actual debt ratio of the firms (*LVB*) is greater than the target debt ratio (LVB*t), the group is classified as over-indebtedness; otherwise, the group is classified as non-over-indebtedness.

The results of the tests for debt heterogeneity are shown in Table 6. In the over-indebtedness group, *FIN_SQ* is negatively related to *TFP_OP* and *TFP_LP* at 1% significance level, which is consistent with the benchmark test; in the non-over-indebtedness group, *FIN_SQ* is not significantly related to *TFP_OP* and *TFP_LP*, which indicates that over-indebted firms, in order to hedge their profits or repay their debts, are more inclined to invest internal debt funds in financial assets, which ultimately creates a disincentive for enterprise to upgrade.

**Table 6:** Test for heterogeneity of liabilities.

Variable	Over-indebtedness		Non-over-indebtedness	
	*TFP_OP*	*TFP_LP*	*TFP_OP*	*TFP_LP*
*FIN*	0.709^**^	0.694^**^	0.264	0.307
	(2.390)	(2.303)	(1.234)	(1.453)
*FIN_SQ*	−1.461^***^ (−3.044)	−1.430^***^	−0.400 (−1.274)	−0.435
		(−2.924)		(−1.404)
*Control*	YES	YES	YES	YES
*η/μ*	YES	YES	YES	YES
*_cons*	−1.184^***^	−1.291^***^	−1.908^***^	−2.006^***^
	(−3.857)	(−4.213)	(−5.908)	(−6.255)
*Adj R*^*2*^	0.767	0.771	0.786	0.792
*N*	2235	2235	2398	2398

***, **, * represents significance at the 1%, 5%, and 10% levels, respectively, with Z-values in parentheses.

#### Property heterogeneity test

When studying the financial issues of listed companies in China’s unique system, it is essential to recognize the critical role played by the nature of property rights. It is widely accepted in academic circles that Chinese SOEs, compared to non-SOEs, imply larger asset sizes and better credit conditions. SOEs also have access to political connections through informal mechanisms and can raise funds at relatively low financing costs while accessing credit facilities from banks and other financial institutions (Lu et al., 2012). Therefore, the impact of SOEs’ financial assets on the upgrading of enterprises should be different from that of non-SOEs.

The results of the heterogeneity test for property rights are shown in Table 7. In the SOE group, *FIN_SQ* is not significantly related to both *TFP_OP* and *TFP_LP*; in contrast, in the non-SOE group, *FIN_SQ* is negatively related to both *TFP_OP* and *TFP_LP* at the 1% significance level, indicating that the inverse U relationship between financial assets and enterprise upgrading is significant only for non-SOEs. Chinese non-SOE listed firms tend to have smaller asset sizes and lower cash flow, more sensitive to investment in financial assets. When firms’ financial assets increase beyond their predetermined thresholds, cash flow becomes difficult to turnover, leading to lower productivity and ultimately hindering enterprise upgrading.

**Table 7:** Test for heterogeneity of property rights.

Variable	State-owned enterprises		Non-state-owned enterprises	
	*TFP_OP*	*TFP_LP*	*TFP_OP*	*TFP_LP*
*FIN*	0.144	0.159	0.287^*^	0.310^**^
	(0.561)	(0.623)	(1.817)	(1.964)
*FIN_SQ*	−0.298 (−0.766)	−0.313	−0.594^***^ (−2.975)	−0.609^***^
		(−0.815)		(−3.062)
*Control*	YES	YES	YES	YES
*η/μ*	YES	YES	YES	YES
*_cons*	−0.811^***^	−0.906^***^	−0.620^**^	−0.735^***^
	(−2.768)	(−3.119)	(−2.295)	(−2.703)
*Adj R*^*2*^	0.789	0.792	0.751	0.754
*N*	3947	3947	5693	5693

***, **, * represents significance at the 1%, 5%, and 10% levels, respectively, with Z-values in parentheses.

#### Financing heterogeneity test

Ease of access to external finance is one of the fundamental reasons why firms invest in financial assets or not. When financing constraints, firms tend to save some capital in advance to avoid the risk of cash flow disruptions. When financing constraints are more severe, firms tend to invest this capital in financial assets rather than physical assets because they hope the high returns from financial assets can alleviate the external liquidity crisis. In this study, the *KZ* index of the same industry in the same year is selected as a proxy variable for the financing constraint of enterprises. If the actual financing constraint of enterprises (*KZ*) is larger than the target debt ratio (KZ*t), it is set as the high financing constraint group; otherwise, it is set as the low financing constraint group (Buch et al., 2014).

The results of the financing heterogeneity test are shown in Table 8. In the high financing constraint group, *FIN_SQ* is negatively related to *TFP_OP* and *TFP_LP* at the 1% significance level, which is consistent with the benchmark results. In contrast, in the low financing constraint group, *FIN_SQ* is not significantly related to *TFP_OP* and *TFP_LP*, suggesting that, compared to low financing constraint firms, high financing constraint firms are more inclined to invest funds in financial assets in the hope of exchanging them for higher profits. However, investing too much in financial assets will exacerbate firms’ liquidity crisis and ultimately inhibit the enterprise’s upgrading.

**Table 8:** Test for financing heterogeneity.

Variable	High financing constraints		Low financing constraints	
	*TFP_OP*	*TFP_LP*	*TFP_OP*	*TFP_LP*
*FIN*	0.530^**^	0.515^**^	0.140	0.195
	(2.556)	(2.455)	(0.779)	(1.086)
*FIN_SQ*	−0.948^***^ (−3.100)	−0.916^***^	−0.300 (−1.229)	−0.348
		(−2.924)		(−1.436)
*Control*	YES	YES	YES	YES
*η/μ*	YES	YES	YES	YES
*_cons*	−1.337^***^	−1.441^***^	−1.062^***^	−1.155^***^
	(−5.531)	(−5.962)	(−4.397)	(−4.791)
*Adj R*^*2*^	0.776	0.780	0.789	0.794
*N*	4582	4582	4612	4612

***, **, * represents significance at the 1%, 5%, and 10% levels, respectively, with Z-values in parentheses.

#### Moderating effect test

The previous estimation results have shown that financial assets significantly impact the enterprise upgrading of Chinese listed companies. In this part, the moderating effect through which financial assets influence enterprise upgrading will be further analyzed. Effectively balancing supply and demand, cost and revenue, and optimizing asset structure is critical to a company’s daily production and operations. However, in China’s capital market, the cost of external financing from the market is much higher than the cost of internal financing. The resulting difference in the cost of internal and external financing leads to a gradual substitution between financial and physical investment, i.e., physical investment not only has a “crowding out” effect on investment in financial assets but also mitigates the marginal disincentive effect of financial assets on enterprise upgrading. However, it is crucial to recognize that firms prefer to invest their limited funds in financial assets rather than in real assets because they can generate short-term excess returns as quickly as possible. This aggravates the degree of capital mismatch, reduces the efficiency of production factor allocation, and exacerbates the marginal disincentive effect of financial assets on enterprise upgrading.

**Table 9:** Tests of the moderating effect.

Panel A	*TFP_OP*	*TFP_LP*
*FIN*	3.766^***^	3.647^***^
	(3.318)	(3.211)
*FIN_SQ*	−3.786^***^	−3.626^**^
	(−2.606)	(−2.484)
*CAP*	−0.028^**^	−0.028^**^
	(−2.359)	(−2.356)
FIN*CAP	−0.185^***^	−0.178^***^
	(−2.956)	(−2.831)
FIN_SQ*CAP	0.167^**^	0.157^*^
	(1.993)	(1.867)
*Control*	YES	YES
*η/μ*	YES	YES
*_cons*	−1.798^***^	−1.874^***^
	(−7.261)	(−7.569)
*adj R*^*2*^	0.780	0.785
*N*	9323	9323
**Panel B**	***TFP_OP***	***TFP_LP***
*FIN*	0.331^**^	0.351^**^
	(2.390)	(2.522)
*FIN_SQ*	−0.575^***^	−0.590^***^
	(−3.163)	(−3.235)
*CAP*	−0.025^**^	−0.024^**^
	(−2.137)	(−1.978)
FIN*CAP	0.168^**^	0.164^**^
	(2.222)	(2.109)
FIN_SQ*CAP	−0.249^***^	−0.239^**^
	(−2.825)	(−2.563)
*Control*	YES	YES
*η/μ*	YES	YES
*_cons*	−1.177^***^	−1.281^***^
	(−6.458)	(−7.034)
*Adj R*^*2*^	0.778	0.783
*N*	9635	9635

***, **, * represents significance at the 1%, 5%, and 10% levels, respectively, with Z-values in parentheses.

To test the possible moderating effect of financial assets in influencing enterprise upgrading, we construct the model as follows (Acharya et al., 2019):

8 $$\begin{array}{l}TEP_ - OP_{j,t}\left( {TFP_ - LP_{j,t}} \right) = \alpha _0 + \alpha _1FIN_{j,t} + \alpha _2FIN_ - SQ_{j,t} \\\qquad\qquad\qquad\qquad\qquad\quad + \alpha _3CAP_{j,t}\left( {RF_{j,t}} \right) + \alpha _4FIN_{j,t} \ast CAP_{j,t}\left( {RF_{j,t}} \right)\\ \qquad\qquad\qquad\qquad\qquad\quad + \alpha _5FIN_ - SQ_{j,t} \ast CAP_{j,t}\left( {RF_{j,t}} \right)\\\qquad\qquad\qquad\qquad\qquad\quad + \gamma {\it{Control}}_{j,t} + \Sigma \eta _{j,t} + \Sigma \mu _{j,t} + \varepsilon _{j,t}\end{array}$$

The degree of capital expenditure (*Cap*) is chosen as a proxy variable for capital investment in business entities. It is calculated as *LN(cash paid for the purchase and construction of fixed assets, intangible assets, and other long-term assets)*. Financial channel profitability is a proxy variable for financial asset allocation gain (*RF*). It is calculated as *(investment income* + *Profit and Loss from Fair Value Changes* + *Net exchange gains and losses* *–* *investment income from associates or joint ventures* *–* *operating profit)/absolute value of operating profit*.

The main effect of the moderator variable on the inverted “U” curve is that it changes the slope of the curve at both ends and shifts the inflection point of the curve. According to the calculation, if *α*_5_ < 0, the moderator variable makes the inverted “U” curve steeper, i.e., the moderator variable reduces the slope of the curve^2^

9 $$\begin{array}{*{20}{c}} {K = TEP_ - OP_{j,t}\left( {TFP_ - LP_{j,t}} \right)^\prime\prime = 2\alpha _2 + 2\alpha _5CAP_{j,t}\left( {RF_{j,t}} \right)} \end{array}$$

The partial derivative of equation (9) gives:

10 $$\begin{array}{*{20}{c}} {\frac{{\partial K}}{{\partial CAP_{j,t}\left( {RF_{j,t}} \right)}} = 2\alpha _5} \end{array}$$

If *α*_1_*α*_5 _− *α*_2_*α*_4_ > 0, it means that the moderator variable shifts the inflection point of the inverted “U” curve to the right^3^

11 $$\begin{array}{*{20}{c}} {\partial FIN_{j,t}^ \ast = \frac{{ - \alpha _1 - \alpha _4CAP_{j,t}\left( {RF_{j,t}} \right)}}{{2\alpha _2 + 2\alpha _5CAP_{j,t}\left( {RF_{j,t}} \right)}}} \end{array}$$

The partial derivative of the inflection points of equation (11):

12 $$\begin{array}{*{20}{c}} {\frac{{\partial FIN_{j,t}^ \ast }}{{\partial CAP_{j,t}\left( {RF_{j,t}} \right)}} = \frac{{\alpha _1\alpha _5 - \alpha _2\alpha _4}}{{2\left( {\alpha _2 + \alpha _5CAP_{j,t}\left( {RF_{j,t}} \right)} \right)^2}}}. \end{array}$$

The results of the test for moderating effects are shown in Table 9. In Panel A, FIN_SQ*CAP and *TFP_OP* are positively correlated with *TFP_LP* at least at the 10% significance level, indicating that the degree of capital expenditure can smooth the nonlinear relationship between financial assets and enterprise upgrading, making the inverted “U” curve flatter; the partial derivative at the curve’s inflection point is <0, indicating that the inflection point shifting to the left. In Panel B, FIN_SQ*RF and *TFP_OP* are negatively correlated with *TFP_LP* at least at the 5% significance level, indicating that the degree of profitability of the financial channel exacerbates the nonlinear relationship between financial assets and enterprise upgrading, making the inverted “U” curve steeper; the partial derivative at the curve’s inflection point is >0, indicating that inflection point shifting to the right.

## Discussions and conclusions

China’s economic growth is undergoing a strategic transition from factor-driven growth to innovation-driven growth. This structural change has gradually revealed tensions between the supply and demand of capital in China’s financial system and the real economy. How will substantial financial assets impact the enterprise upgrading of Chinese A-share listed companies? Our analysis finds:(1) Short-term financial assets significantly enable enterprise upgrading. (2) Long-term financial assets significantly inhibit enterprise upgrading. (3) The relationship between financial asset allocation and enterprise upgrading resembles an inverted U-shape with nonlinear effects. (4) The risk-taking capacity of short-term financial assets is a crucial mechanism influencing the enterprise upgrading of Chinese non-financial A-share listed companies. (5) The earnings persistence of long-term financial assets is a vital mechanism inhibiting the enterprise upgrading of Chinese non-financial A-share listed companies. (6) Financial assets can impact the enterprise upgrading of over-indebted, non-state-owned, and highly constrained Chinese non-financial A-share listed companies but not significantly affect the enterprise upgrading of non-over-indebted, state-owned, and low-constraint Chinese non-financial A-share listed companies. (7) Capital expenditures can smooth the inverted U-shaped relationship between financial assets and enterprise upgrading. (8) The profitability of financial channels can intensify the inverted U-shaped relationship between financial assets and enterprise upgrading.

This study’s conclusions also imply the following policy recommendations:(1) Optimize corporate capital allocation channels and promptly adjust investment structures. Given the dual nature of financial asset investments, enterprises should dynamically optimize investment behavior management mechanisms while managing asset structures and investing in financial assets. They should leverage the advantages of short-term financial assets and avoid overinvesting in long-term financial assets according to business objectives and cash flow positions. This will ensure asset preservation and avoid profit-seeking traps, preventing financial assets from depleting physical production. (2) Strengthen securities regulations to curb market speculation. On the one hand, securities regulators should establish dynamic, real-time, and comprehensive regulatory systems to detect and prevent the excessive expansion of virtual economies and illegal arbitrage in capital markets. On the other hand, they should improve the quality and disclosure duration requirements for listed companies, balance information asymmetry among listed companies, investors, and regulators, and prevent companies from masking financial gains by disclosing negative information. (3) Expand financing channels for real assets and optimize the symbiotic relationship between industry and finance. This involves enhancing banks’ ability to provide continuous credit, ensuring the standardized development of bond markets, and meeting enterprises’ reasonable capital needs for physical production through convenient financing channels. This would prevent excessive reliance on long-term financial assets and high-risk speculative behavior.

Despite the valuable implications obtained from the results, our study has some limitations. Firstly, the fact that our study only focuses on firms in China limits the generalizability of our findings. Future research should conduct cross-national analyses to test the robustness of our results in other economic entities. Secondly, the enterprise upgrading measurement of firm growth capability may require revision and further testing and refinement. Future empirical research can adopt field research and case studies to capture facets of the enterprise’s upgrade capability. Thirdly, the regression analysis and mediation effect test requirements for parameter estimation are insufficient compared to China’s large population and numerous enterprises. Therefore, it is necessary to increase the sample size in future studies. Lastly, although we have controlled for firm age, size, and industry, these variables may not encompass all possible contextual differences that have influenced the relationships studied in our conceptual models. Therefore, future studies should focus on other potentially significant control variables.

Notwithstanding the abovementioned limitations, our study presents some possible future research directions. For instance, R&D investment and the profit-to-cost ratio can be used as mediators to explore further the outside-in mechanism of the promotion of enterprise upgrading. Scholars can also verify our findings from other perspectives, such as examining the relationship between financial assets and enterprise upgrading using data from the Chinese industrial enterprise database.

## Data Availability

The datasets used or analyzed during the current study are available from the corresponding author upon reasonable request. Further data is publically available on China Securities Markets and Accounting Research Databases.

## Footnotes

1 $TEP_ - OP_{j,t}(TFP_ - LP_{j,t})$ taked partial derivative of *FIN*_*j,t*_ to obtain $\frac{{TEP_ - OP_{j,t}(TFP_ - LP_{j,t})}}{{FIN_{j,t}}} = \alpha _1 + \alpha _2FIN_{j,t}$, and make it zero, then the inflection point as −*α*_1_/2*α*_2_.

2 The slope of the inverted “U” curve is calculated as follows: the second-order partial derivative of *FIN*_*j,t*_ for Eq. (8) yields the vertex slope K:

3 The inflection point of the inverted “U” curve is calculated as follows: For Eq. (8) *FIN*_*j,t*_ find the first-order partial derivative and make it equal to 0:
